# Lectures on Diseases of the Skin

**Published:** 1887-05

**Authors:** Henry Wile

**Affiliations:** Lecturer on Dermatology, Atlanta, Ga.


THE ATLANTA MEDICAL AND SURGICAL JOURNAL.

VOL. IV. MAY, 1887. No. 3.

### Original Communications.

### LECTURES ON DISEASES OF THE SKIN.

DELIVERED AT THE ATLANTA MEDICAL COLLEGE DURING THE SESSION OF 1885-86, BY HENRY WILE, M. D., LECTURER ON DERMATOLOGY, ATLANTA, GA.

### LECTURE XIV.

### HERPES ZOSTER—MILIARIA—POMPHOLYX—PEMPHIGUS—LICHEN RUBER.

Reported Stenographically for THE ATLANTA MEDICAL AND SURGICAL JOURNAL by W. Kay Tewksbury.

HERPES ZOSTER is a cutaneous disease running an acute inflammatory course characterized by an eruption of vesicles and the presence of pain. The pain is neuralgic, generally forming a marked prodromal symptom. It may manifest itself in different localities, but always in the course of the cutaneous nerves. This symptom continues a few days when the skin becomes red, and on this red surface the eruption makes its appearance in the form of variously sized groups of pin-head sized vesicles. These come out in successive crops and during their existence, manifesting a disposition to remain intact and not to rupture, as in vesicular eczema. The vesicles may remain stationary for three or four days, or may develop and coalesce forming blebs. The lesions dry up, leaving yellowish crusts. About the eighth or tenth day the eruption is at its height, presenting at that time a characteristic appearance, the lesions being in different stages of developement situated on a bright erythematous base, and situated along the tracks of the nerves, appearing in rows or streaks. At the end of the twelfth or fourteenth day the eruption disappears altogether, leaving no trace except where the process has been intense, in which case ulcerations may ensue and continue for several weeks, and disappearing leave scars. The neuralgic pain varies in its intensity, but is often so severe as to keep the patient awake at night and prove a source of great distress.

The disease is subdivided, according to its location, into varieties which take their names from the nerve of the part affected. Thus we have herpes capitis, frontalis, facialis, cervicalis, cervico brachialis, brachialis, dorso-pectoralis, lumbo abdominalis, lumbo-femoralis, etc., appearing along both the cranial and the spinal nerves.

Herpes Zoster of the face is usually severe. It appears here along the course of the branches of the trigeminus nerve, which is the sensory nerve of the face, and is analogous to a spinal nerve, in that it has on its posterior root a ganglion. When the disease occurs along the tract of the ophthalmic nerve, one of the branches, it makes its appearance usually on the forehead over the eyes, to which part this nerve sends fibres. The eruption, when it occurs here, may be attended with grave consequences to the eye, such as ulceration of the cornea, suppuration of the eye-ball, even extension of the ulceration to the brain resulting fatally. The typical form of herpes zoster is where it appears along the course of the intercostal nerves, occurring in streaks, occupying the walls of the chest up to the median line, by which the lesions are sharply limited. It is, as a rule, uni-lateral, and seldom attacks both sides of the body. It is, as a rule, a disease of adult life, and generally attacks a person but once. Neuralgia may be a sequela, especially on the face.

The cause of the disease is attributed to influences communicated to certain nerve centres through their peripheral terminations; thus sudden exposure, traumatism. An illustration of the former I presented to you a few weeks ago. A young man, after participating in the festivities of a skating rink, and, as he said, being in a state of profuse perspiration, went out into the winter night air. After a few hours he experienced sharp neuralgic pains in the right lumbar region, also some darting down the right leg. After three days the characteristic eruption appeared along the lumbo-abdomino femoral nerves.

The process is essentially an inflammation of the sheath of the cutaneous nerves, the neurolemma.

The eruption is secondary to this neuritis and consists in a vesicular inflammation of the skin. The vesicle of herpes zoster, which starts in the deep layers of the rete, is divided into compartments, the partitions consisting of thin layers of epidermal cells forced apart by the exudation. The cells composing the partition are spindle-shaped. The blood-vessels of the cutis are dilated and congested and surrounded with lymphoid cells. The nerve-sheath is also said to be the seat of cellular infiltration. In the casserian ganglion, on the side on which the disease occurs, one authority, Bärensprung, pointed out certain changes consisting of hemorrhage and fatty degeneration of the ganglion cells.

The prognosis is generally favorable. The disease disappears without leaving any trace except where it has been intense and the surface has been lacerated by scratching. In the latter case ulcerations may follow which may prove obstinate to treatment.

The diagnosis presents no difficulty on account of the peculiar and characteristic manifestations of the disease. The only disease with which it may be confounded is vesicular eczema, and it is distinguished from the latter by the fact that it is preceded by and attended with sharp neuralgic pains, and that the vesicles remain intact, whereas the vesicles in eczema rupture very soon after development; besides this the lesions of herpes zoster are confined to certain localities and appear in distinct tracts along the course of the nerves.

The treatment is palliative as the disease is self-limited, although it is claimed by some that electricity in the form of the galvanic or constant current will sometimes abort the attack. It often affords relief from the distressing pain. The current may be applied with five or ten cells, the positive electrode being placed over the region of the spinal cord, while the negative electrode is brushed gently over the seat of the affection. It is usually applied ten to thirty minutes. Local treatment may further consist in the use of soothing lotions or ointments, such as a lotion of calamine and oxide of zinc equal parts, as a drachm to four ounces of rose water with a drachm of glycerine, or applications of oxide of zinc and vaseline spread on muslin rags. If the disease is so distressing as to keep the patient awake at night, morphia may be administered.

MILIARIA is an inflammatory disease of the sweat-glands and their ducts and is characterized by an eruption of papules or vesicles around the outlets of the glands. It is popularly known as prickly heat. It appears especially during the summer season or at any time when the perspiratory apparatus is thrown into a state of great activity. It is common in infants, especially the weak and sickly when they are covered with a superabundance of clothing. The disease is, as a rule, universal and appears in two forms, papular and vesicular, though they are not always distinct, the two varieties appearing commonly together. It occurs mostly in corpulent people in whom the sweat-glands are active. There exists an intense congestion of the glands which brings about an inflammation of the ducts and outlets that results in the development of vesicles and papules.

The only disease for which it may be mistaken is vesicular eczema. The suddenness of its onset, its appearance without warning, the location of the lesions around the outlets of the sweat-glands, the sensation of tingling or burning are the features which distinguish it at once.

The object of treatment is to remove the cause, and it is therefore necessary to advise the patient to keep cool, so as to allow the inflammatory symptoms to subside. Recourse may also be had to cooling applications, such as cologne water or equal parts of alcohol and water, or anything that will produce rapid evaporation resulting in a lowering of the temperature of the part. The same result can be attained by cold sponge baths. Absorbent powders are also useful, such as equal parts of talc, calamine, oxide of zinc and carbonate of magnesia. This is to be dusted on the part frequently during the day. A lotion of sulphate of zinc, from a drachm to a drachm and a half to half a pint of rose water used several times a day, is likewise of value. Under this treatment the disease will subside.

POMPHOLYX is a cutaneous affection, as obscure as it is rare, and it is not yet definitely classified. It appears in the form of vesicles and blebs, especially around the fingers and toes. The disease is also known as DYSIDROSIS, from a supposed implacation of the sweat-glands, that is, a difficulty in the discharge of their function. These vesicles first appear in the deep layers of the skin, and after a time approach the surface and present the appearance of *boiled sago*. If the lesions are pricked with a needle the watery contents oozes out upon the surface. As the lesions reach the surface the vesicles rupture and discharge their contents. This is accompanied by more or less exfoliation of the epidermis. Very few cases of this disease have been recorded.

PEMPHIGUS, a rare disease, is an inflammatory process characterized by the formation of bullæ, or blebs. There are two forms of the disease, *pemphigus vulgaris* and *pemphigus foliaceus*.

*Pemphigus vulgaris*, the more common of the two, appears in the form of blebs varying in size from a small hazel-nut to a goose-egg. The lesions develop quickly, in the course of twenty-four hours, and are usually ushered in by constitutional disturbance, especially chill, fever, loss of appetite. They are generally scattered about in different regions of the body, even upon mucous membranes, and appear in successive crops, also having a tendency to remain intact unless ruptured mechanically by friction of the clothing. After a few days they dry up and form crusts or rupture and form crusts which in turn fall off, leaving slight brownish stains. The blebs are first filled with a clear serous fluid, which may or may not be stained with blood. After they have existed for one or two days, their contents become cloudy. The disease may be acute or chronic—as a rule the latter. Acute pemphigus runs its course in a few weeks or months, the patient recovering entirely. Chronic pemphigus is generally associated with some general debility of the system. Lesions may be continually appearing without intermission, or relapses may occur from time to time. The constant discharge of serum from the blebs is a drain upon the system which finally causes marasmus, to which the patient succumbs.

*Pemphigus foliaceus* exhibits an incomplete development of the lesions. The blebs do not become fully distended with the exudation, but remain flaccid. They become about half filled with the serum and easily rupture. Then occurs an exfoliation of the epidermis which continues until the corium is exposed. This form is chronic, lasting sometimes for years, spreading slowly until large surfaces are involved. Most cases end fatally.

Both forms of this disease are accompanied by itching and burning—the latter often by great pain and distress when large surfaces are involved, and the patient is obliged to remain in bed. The cause of the disease is unknown. It occurs more among males than females, and affects infants more than adults. Heredity has been noticed in a few instances, but that it is contagious has by no means been established. A chemical analysis of the contents of the bleb throws no light upon the nature of the disease, for it reveals nothing more than is found in a blister produced mechanically upon the skin. The lesions of pemphigus are superficial. They are situated between the upper layers of the epidermis, are one-chambered and filled with a clear, later, cloudy serous fluid, containing degenerated epidermal cells, fat crystals and possibly pus and blood. The papillæ of the corium are swollen and crowded with a cellular infiltration, and Neumann has observed a dilatation of the sweat-glands. The process is accompanied by no loss of tissue, a temporary pigmentation alone remaining to mark the site of the lesions.

The diagnosis of the disease presents little difficulty, on account of the size and superficial character of the lesions, the frequency of relapses and the accompanying general constitutional disturbance.

There is a disease occurring in infancy, soon after birth, characterized by a bullous eruption affecting those who are subject to hereditary syphilis. It is therefore known as syphilitic pemphigus. It is not, however, to be classified as a form of pemphigus, but on account of other concomitant symptoms, such as coryza, fissures at the angles of the mouth or about the arms, and the appearance of the lesions, especially about the hands and feet, it is rather to be grouped under the head of syphilitic eruptions.

Pemphigus may resemble *herpes iris*, but the latter disease is at first confined to the region of the hands and feet, and its lesions appearing always upon an erythematous base are arranged in concentric rings. Besides this, in pemphigus relapses are the rule, whereas, in herpes iris the rarest exception. It is distinguished from *erythema bullosum* by the absence of nodules in the skin and rheumatic pain in the joints.

Pemphigus is further to be differentiated from bleb-like eruption that may follow the ingestion of certain drugs. Thus it has been observed after the administration of large doses of iodide of potash. Occasionally, among a class of individuals whose sole object in life seems to be to simulate disease, there will be found one who will feign this affection by producing blebs upon the skin mechanically by means of such agents as ammonia and cantharides. Caustic agents produce more or less inflammation around the lesion which is absent in pemphigus; whereas, microscopical examination of a bit of skin over the seat of the lesion will generally reveal the shining, colored wing of the Spanish fly.

The prognosis is usually favorable in the acute form, but when chronic and the relapses are frequent, with numerous lesions, it is not so bright. In pemphigus foliaceus it is, as a rule, grave.

As to the treatment, it consists of the administration of tonic remedies, such as iron, quinine and arsenic. There is no specific for this disease; such remedies may be administered as are calculated to keep up the strength of the patient. The local treatment is also palliative. The blebs should be carefully ruptured, allowing the contents to escape, thus preventing septic absorption. This also facilitates the disappearance of the lesion. Some absorbent powder may then be used to take up the liquid. Where the disease is very extensive, involving the larger part of the surface of the skin, as in pemphigus foliaceus, the continual water-bed offers the best results. The water must be kept at a uniform temperature and changed at proper times. The surface of the skin soon accommodates itself to its new surroundings, and the patients express themselves as feeling comfortable, specially on account of the marked diminution of the subjective symptoms, such as burning and itching. Baths containing bi-carbonate of soda or corrosive sublimate may also be advised.

*Lichen Ruber*, another one of the rare diseases, appears in the form of pin-head to small, pea-sized, pale red, or copper-colored, glistening, flat or pointed papules. The lesions all have a polygonal base, and the flat ones are slightly umbilicated. According to the form of the lesions, whether pointed or flat, there are two varieties of the disease—*lichen acuminatus* and *lichen planus*. Both varieties often appear together. The eruption may be universal from the start or be confined to some locality. The accuminate variety usually first appears on the trunk, while the plain variety appears generally on the extremities. The disease is progressive. The papules develop slowly, remaining as papules for weeks and then are absorbed, while new crops continue to appear for years, unless treated, until the whole surface may be covered with lesions, especially of the accuminate variety. The papules on being absorbed leave characteristic pigment stains and often scar-like depressions. Lesions may occur upon mucous membranes. The disease runs a mild course in this country, but in Europe it develops more serious characters and often ends fatally.

The cause is unknown. The disease appears between the ages of ten and forty years, and according to the statistics of Kaposi two-thirds of the cases were seen in males and one-third in females. It seems also to be a disease occurring more among b etter classes of society.

These papules, when examined miscroscopically, show a thickening of the epidermal layer and an œdematous condition of the papillæ of the corium. The blood-vessels are dilated and surrounded with proliferating cells. A peculiar feature is the development of the lesions immediately around the hair follicle, and the chief change seems to be around the lower part of the follicle. The outer root sheath presents villous projections, the upper part of the sheath being hypertrophied in the shape of a cone with the joint downward. The cells of the rete Malpighii also appear granular.

